# Adolescents’ and their parents’ experiences of using a closed-loop system to manage type 1 diabetes in everyday life: qualitative study

**DOI:** 10.1177/1742395320985924

**Published:** 2021-01-20

**Authors:** D Rankin, B Kimbell, R Hovorka, J Lawton

**Affiliations:** 1 Usher Institute, University of Edinburgh, Edinburgh, UK; 2 Wellcome Trust – Medical Research Institute of Metabolic Science, University of Cambridge, Cambridge, UK; 3 Department of Paediatrics, University of Cambridge, Cambridge, UK

**Keywords:** Type 1 diabetes, closed-loop systems, qualitative, participant experiences, technology

## Abstract

**Objectives:**

Type 1 diabetes can have life-shattering consequences for adolescents and parents. A closed-loop system is a cutting-edge technology which automatically regulates glucose to reduce the burden of diabetes management. We explored adolescents’ and parents’ experiences of using this technology to understand how it affects their biographies and everyday lives.

**Methods:**

In-depth interviews with 18 adolescents newly diagnosed with type 1 diabetes and 21 parents after ≥12 months experience using closed-loop technology. Data were analysed thematically.

**Results:**

Participants reported very few disruptions to their lives when using a closed-loop. Reports of family conflict were minimal as the closed-loop enabled dietary flexibility and glucose levels to be checked effortlessly. Adolescents described doing ‘normal’ activities without worrying about high/low glucose, and parents reported allowing them to do so unsupervised because the closed-loop would regulate their glucose and keep them safe. Some adolescents expressed concerns about the visibility of components and, to avoid stigma, described curtailing activities such as swimming. Participants described how the closed-loop enabled adolescents to be in control of, or create distance from, diabetes.

**Discussion:**

The closed-loop has life-enhancing consequences for both adolescents and parents and helps to reduce the biographical disruption of type 1 diabetes in this age group.

## Introduction

Type 1 diabetes is one of the commonest chronic conditions in young people.^1^ It results from the destruction of insulin-producing pancreatic beta cells which regulate blood glucose. Hence, individuals need to keep their blood glucose within target range by self-administering insulin, which they adjust according to current glucose levels (often determined through 5–6 times daily finger-prick checks), the carbohydrate content of meals/snacks consumed and physical activity.^2^ When glucose levels are not in range individuals can experience hypoglycaemia (low blood glucose), which includes symptoms such as confusion, altered emotions and, in extreme cases, loss of consciousness, seizure and coma, or hyperglycaemia (high blood glucose), which, in the short-term, can result in life-threatening ketoacidosis and, in the longer-term, can increase risk of cardiovascular complications.

Until recently, most individuals with type 1 diabetes have used multiple daily injection (MDI) or insulin pump regimens. However, the development of an innovative technology called a closed-loop system,^3^ sometimes known as an artificial pancreas, may constitute a potential sea-change in how type 1 diabetes is managed and experienced. The closed-loop system combines a real-time continuous glucose monitor (CGM) with an insulin pump and a computer-based algorithm which translates, in real-time, glucose information from the CGM to compute the amount of insulin delivered by the pump. By automatically regulating insulin delivery, an intended purpose of this technology is to reduce the burden of diabetes self-management and improve quality-of-life.^4^

Adolescence is a demanding developmental stage with particular challenges experienced by those with type 1 diabetes.^5^ Physiological changes coupled with the complex requirements of managing this chronic condition often result in individuals in this age group having suboptimal glucose control, higher than recommended levels of glycosylated haemoglobin (HbA1c) (an average measure of blood glucose control),^6^ and high levels of distress, anxiety, depression, difficulties coping and low self-esteem.^7^ A growing body of research has sought to understand the distinctive challenges encountered by individuals in this age group. This research has predominantly focused on those using MDI or insulin pump regimens to manage their diabetes and their parents/caregivers (e.g.^8–10^) Four major areas emerge from this literature.

### Family conflict

Type 1 diabetes can have a negative impact on family life that often manifests in conflicts between adolescents and parents, particularly around food and eating. This includes disagreements about sugary foods which adolescents often want to consume, but parents seek to restrict because of worries about high blood glucose which, over time, can increase risk of long-term complications.^8,9,11^ Family conflict can also result from parents feeling they need constantly to remind and prompt their child to monitor their blood glucose using finger-prick checks while youths resent being ‘nagged’ to do so.^9,12–14^

### Wanting to be normal and neglecting self-management tasks

Adolescents describe resenting having to perform diabetes management tasks (e.g. checking blood glucose, administering insulin) when these interfere with or curtail social activities and make them feel different to their peers.^8,15,16^ Hence, adolescents may neglect self-management tasks in order to participate in social activities and fit in with others.^8,10,12,13,15^

### Fear of hypoglycaemia and concerns about complications

Fear of hypoglycaemia is a major concern for adolescents, who report intentionally elevating blood glucose levels in order to feel safe (e.g. when socialising with peers, before going to bed).^12,14,16,17^ Similar concerns have been reported by parents, who describe feeling anxious and distressed that their child might not be able to treat hypoglycaemia successfully on their own.^12,14,18^ Hence, parents describe feeling constantly worried and getting up often during the night to perform blood glucose checks to help ensure their child’s safety.^12,14,18,19^

### Adapting to diabetes

Studies indicate that there are two main styles of adapting to diabetes. While some adolescents attempt to integrate diabetes into their lifestyle and prioritise self-management, others reject diabetes and neglect carrying out diabetes-related tasks.^10,13,15^

The above literature offers important insights by drawing attention to the complex and multifaceted ways in which type 1 diabetes can affect the everyday lives of adolescents and their families. Indeed, because of its far-reaching impact, some commentators have suggested that the onset and management of type 1 diabetes needs to be understood as a biographically disruptive event,^20^ which undermines the taken-for-granted fabric of one’s existence and self-concepts (such being a ‘normal’ teenager), and requires significant adjustments and mobilization of resources to be made.^15,21^

While the biographically disruptive impact of type 1 diabetes is now well established, very little is known about adolescents’ and parents’ experiences of using newer technologies such as closed-loop systems, which, as indicated above, aim to reduce the burden of self-management tasks and improve users’ quality of life. To date, studies have explored adolescents’ and family members’ experiences of using closed-loops for short durations or as part of larger samples predominantly comprising adults;^22–27^ hence, there have been calls for longer studies to be undertaken with adolescents in real-life conditions.^23^ In this paper, we report findings from an interview study involving adolescents and their parents who participated in the Closed Loop from Onset in type 1 Diabetes (CLOuD) trial.^28^ This UK-based, open-label, multi-centre trial explored the clinical and other benefits of a day-and-night hybrid closed-loop system as compared to a multiple daily injection (MDI) regimen in young people (aged 10–16.9 years) newly diagnosed with type 1 diabetes. Participants were randomly assigned to 24 months of study intervention, followed by an optional 24-month extension phase (see Table 1).

**Table 1.** Description of the Closed Loop from Onset in type 1 Diabetes (CLOuD) trial, closed-loop system and trial procedures.

*Trial description and closed-loop system*The CLOuD trial was designed to assess whether closed-loop technology can preserve the function of beta cells in young people who have recently been diagnosed with type 1 diabetes better than standard treatment (MDI).^28^ Recruited participants (n = 96) were randomly assigned to the intervention (closed-loop) or control (MDI) arm of the trial. Participants had to be: aged 10–16.9 years; diagnosed with type 1 diabetes within the previous 21 days; willing to perform capillary blood glucose monitoring (at least 4 blood glucose measurements each day); wear study equipment (glucose sensor and closed-loop system); and, upload pump and CGM data at regular intervals. The automated hybrid closed-loop system used by participants who took part in interviews included: • Modified next generation sensor augmented Medtronic insulin pump 640G (Medtronic Minimed, CA, USA) with CGM receiver and pump suspend feature. • Medtronic CGM Transmitter with Guardian 3 sensor. • An Android smartphone handset containing the Cambridge model predictive control algorithm with a propriety translator to allow wireless communication with the insulin pump. *Training and staff contact received during the trial*The study included up to 14 visits and 1 telephone/email contact for participants completing the trial. During the run-in period, participants were recruited then consented to the trial and asked to complete baseline measures. Participants randomised to the closed-loop group attended the clinical research facility / usual clinic to set up the system over three visits, including: insulin pump training and initiation (three- to four-hours), CGM training and initiation (two hours) and closed-loop initiation (three- to four-hours). Training was provided on initiation and discontinuation of the hybrid closed-loop system, switching between closed-loop and standard insulin pump therapy, meal bolus procedure, and the use of study devices during exercise. A closed-loop system user manual including a trouble-shooting section was handed out during training. Competency on the use of the closed-loop system was assessed. Participants were contacted within 1 week after the initiation of study treatment to review use of study devices. During the two years of the intervention, participants were seen in the clinic at three-month intervals. All participants were provided with a 24-hour helpline to contact the study team in the event of study-related issues.

The closed-loop investigated in this trial comprised two body-mounted devices: an insulin pump the size of a small mobile phone, which attached to the young person’s body using a tube and cannula inserted around the belly-button area, and a CGM subcutaneous sensor with wireless transmitter attached in a similar location. Users were given instructions to refill the pump reservoir and change the infusion set every two to three days, and replace and calibrate the CGM sensor at least every seven days. The machine-learning control algorithm resided in a separate hand-held device (an Android smartphone) which the young person needed to keep in close-proximity (5–10 metres) to avoid signal loss with the pump/CGM receiver. The system also required users to: enter information about carbohydrates consumed (in meals/snacks) so an appropriate dose of extra insulin could be administered by the closed-loop; perform four finger-pricks per day before meals; and, respond to alarms alerting high/low glucose levels or the need to recalibrate the sensor. On the pump, users had access to their sensor glucose reading, glucose trend arrows, active insulin on-board and a graph depicting their sensor glucose profile (real-time glucose levels). On the handset, users could view a graph depicting their sensor glucose profile, insulin administered by the closed-loop, glucose target range indicator line, i.e. their optimum target range, low glucose suspend indicator, and meal and insulin bolus data. Our aim was to explore adolescents’ and their parents’ experiences of using a closed-loop system to better understand how this technology affects the biographies and everyday lives of individuals in this age group newly diagnosed with type 1 diabetes.

## Methods

Qualitative methods are used when little is known about the area under investigation as they allow findings to emerge from the data rather than testing pre-determined hypotheses.^29^ The study was guided by the general principles of Grounded Theory research which advocates a flexible, open-ended approach^30^ and informed by an epistemological position which recognises that self-management practices and engagement with new technologies may be influenced by personal and contextual factors.^31^ Semi-structured interviews were undertaken using a topic guide which included a list of areas to be covered, rather than a set of pre-determined, structured questions. This ensured that discussions remained relevant to addressing the study aims while allowing flexibility for participants to raise issues they perceived to be salient, including those unforeseen at the outset.^30^

### Recruitment and data collection

Adolescents randomised to a closed-loop system and their parents/caregivers were recruited to the interview study by trial staff in six UK sites using an opt-in procedure. Each provided written informed consent. A decision was taken not to approach participants in the MDI arm because our literature review identified multiple studies which offered detailed insight into adolescents’ and parents’ experiences of using MDI and pump regimens in everyday life; hence, it was concluded, no further primary research was necessary. Purposive sampling was used to ensure diversity by taking into account adolescents’ gender, age and parental occupation (as a proxy for socio-economic status). Recruitment was stopped when data saturation occurred. The study received approval from Cambridge East Research Ethics Committee (REC ref: 16/EE/0286).

Interviews took place 12 months after adolescents had commenced use of the closed-loop to allow time for the technology to be embedded in everyday (family) life. Data collection and analysis took place concurrently enabling findings identified in early interviews to iteratively inform areas explored in subsequent accounts.^30^ Interviews were conducted by DR, an experienced non-clinical qualitative researcher between February 2018 and July 2019. The topic guide was informed by literature reviews (including the qualitative research outlined above), input from clinical co-investigators, consultations with patient representatives and revised in light of findings emerging from the first 5 interviews (see Table 2). Adolescents and parents were interviewed separately at a time of their choosing. Interviews lasted between 45–120 minutes, were audio-recorded and transcribed in full.

**Table 2.** Relevant areas explored in interviews.

• Background information: who the adolescent participant lives with, everyday school and family life.
• Adolescents’ and parents’ level of involvement in managing diabetes when using the closed-loop at and away from home (e.g. when cared for by others, at school).
○ Counting carbohydrate in food, determining and administering insulin doses.
○ Monitoring and responding to readings from the CGM sensor; frequency of finger-pricking to monitor blood glucose.
○ Inserting cannulas and CGM sensors, calibrating the CGM, charging the phone handset, ensuring handset and pump are connected, responding to system alarms.
○ Using data on phone handset to inform decisions about insulin administration or to adjust settings on the closed-loop.
• Experiences of managing diabetes using a closed-loop system in everyday life at and away from home (e.g. at school, with friends).
○ Perceptions and understandings of how the closed-loop system worked and how it affected one’s (or one’s child’s) blood glucose control, including: approaches to managing and/or preventing hypoglycaemia or hyperglycaemia; use of corrective doses.
○ Perceived impact of using a closed-loop on food choices and eating practices (meals and snacks).
○ Perceived benefits and burdens of using a closed-loop when undertaking physical activity.
○ Views about the impact of using a closed-loop on one’s (or one’s child’s) social life and independence.
○ Perceived impact of using closed-loop technology on self-perceptions, relationships with others and everyday life.
○ Worries and concerns about having diabetes.
• Any other issues the participant would like to discuss.

### Data analysis

Data were analysed by DR and JL using a thematic approach informed by the method of constant comparison.^30^ Individual interviews were read through repeatedly before being cross-compared to identify recurrent themes. DR and JL undertook separate analyses and wrote separate reports before meeting on several occasions to compare interpretations and develop a coding framework which captured key findings and contextual data needed to aid data interpretation. Data were coded using Nvivo11 (QSR International, Doncaster, Australia). Coded datasets were subjected to further analyses to allow more nuanced interpretations to be developed. To safeguard anonymity, pseudonyms are used in our reporting below.

## Results

The sample comprised of 18 adolescents and 21 parents. Demographic data are presented in Table 3. Below, our results are structured to illustrate how using the closed-loop impacted adolescents’ and parents’ approaches to diabetes management and everyday lives in line with key areas identified in the literature review above. As all the main findings cut across the sample, our reporting has not been separated out according to individual characteristics, such as gender or age.

**Table 3.** Demographic characteristics of study participants.

Characteristic	N	%	Mean ± SD & range
Adolescents (n = 18)			
Female	9	50.0	
Age at time of interview – all children (years)			13.9 ± 1.7, range 11–17
Parents of adolescents (n = 21)			
Female	15	71.5	
Age at time of interview – all parents (years)			46.6 ± 5.3, range 36–56
Occupation^a^				
Professional	12	57.1		
Semi-skilled	7	33.3		
Unemployed/Full-time carer	2	9.5		

^a^Percentages do not sum to 100% due to rounding.

### Family conflict

#### Little or no family conflict about food choices and finger-pricking

Participants did not generally describe experiencing diet-related family conflict when using the closed-loop and none of the adolescents reported parents imposing restrictions on eating carbohydrate-rich, sugary foods. Rather, youths described enjoying a wide range of foods both before and after diagnosis, and making dietary choices without parental interference: “So they’re not saying: ‘well, only eat this many carbs a day’ … or: ‘you have to take packed lunch into school every day’. Or, things like that. They’re not too strict about things like that.” (Clare_13 yrs).

In keeping with adolescents’ accounts, parents described having few concerns about their child’s food choices because they felt reassured that, if the young person miscalculated or forgot to enter information about the amount of carbohydrates consumed, or chose sugary options, the closed-loop would automatically adjust insulin delivery to correct rises (or falls) in glucose levels:

It does give you such tremendous peace of mind that you know it’s working in the background to keep him as steady as possible. And when you look at it [graph on handset] and you can see, where he’s maybe miscalculated, or he’s had a lot of carbs or something and he’s gone really quite high and the background insulin just goes crazy and then brings it straight back down again. (Sue)

Additionally, adolescents did not report having disagreements with their parents about doing finger-pricks to monitor their blood glucose. As Georgia (15 yrs) pointed out, having access to her glucose levels on the handset reduced the need to perform such checks: “say if I want a snack, I don’t have to like keep on pricking my finger. I can literally just check my phone”. While some parents reported needing to prompt their child to do finger-prick checks before bed or to calibrate the device, most, like Rachel, described feeling reassured by the accuracy of the CGM if her daughter did not perform these checks at other times: “I don’t think she probably does any finger-pricks at school unless she’s going low, or an alarm beeps. I could be wrong but equally she’s got a monitor on. It’s pretty accurate.”

#### Parental prompts to ensure the closed-loop works effectively

Whilst diabetes-related family conflicts were seldom reported, parents still described having to prompt or cajole their child to perform various practical tasks to ensure the system was used optimally. For example, several reported routinely having to remind or nag their child to do tasks, including: ensuring that they kept the handset in close proximity; charging the handset; and, calibrating and replacing the CGM sensor. In one example, Karen discussed how her son had struggled to adapt to having diabetes and neglected to perform many of these tasks, which, she reported, resulted in her constantly feeling she needed to be “on his back”:

So there’s a lot of us asking him all the time: ‘has he done something. Has he done this? Has he done that? Has he charged the CLOuD phone?’ (laughs) You know, it’s a lot of things to remember. And I’m sure he feels that we’re on his back, on his case all the time.

### Leading a normal life and fitting in with peers

Adolescents also discussed how using component parts of the system had helped them continue to lead normal lives despite having diabetes. Emma (14 yrs), for instance, who was very physically active, described benefiting from using the CGM because she did not have to interrupt what she was doing to undertake painful and invasive finger-prick checks: “if I’m dancing I can just check it [pump] to see where me bloods are. So it’s not like I’m constantly checking and having to sit out of [stop doing] dance and them kind of things”. Similarly, several adolescents reported how using an insulin pump had helped normalise their lives because they did not need to perform painful injections but instead could administer insulin quickly and discretely in front of others, as they only needed to input the amount of carbohydrate consumed and press a button.

As well as the quality of life benefits derived from using the CGM and pump components of the closed-loop, adolescents described benefiting from the system’s ability to automatically adjust insulin delivery in response to high/low glucose resulting from (unplanned) physical activity and/or errors in carbohydrate counting. This allowed them to get involved in spontaneous games of football or run around with friends in the school playground without worrying about their glucose levels rising and to avoid any potential embarrassment related to symptoms of ‘going low’, such as becoming confused or collapsing in front of others:

I can still do things with this [closed-loop]. I was out at like this youth club that I go to and we went to Laser Tag, and I was asking myself, can I do this with diabetes? And it’s fine … I feel like it’s [closed-loop] just like making sure that my bloods are level and stuff, and like they don’t shoot up or shoot down. (Simon 17 yrs)

Most parents also described feeling sufficiently confident and reassured by the closed-loop’s ability to regulate their child’s glucose levels to allow them to socialise with friends and spend time away from home (e.g. for sleepovers). This included Katie, who contrasted her experiences with those of parents whose children used MDI regimens and who, she said, had “talked about not letting their kids go away and stuff”. As she explained, her confidence in the closed-loop had meant that her son’s social life had continued uninterrupted following diagnosis:

the closed-loop keeps his blood sugars so steady … he still goes to his friends for a sleepover, he’ll go to his grandma’s for a sleepover. You know, nothing’s changed from that point of view. And again, my reliance on the closed-loop and my sort of confidence in that is a big part of that. (Katie)

Similarly, Iris described allowing her son to socialise with friends unsupervised, because, after observing how the closed-loop had performed on previous occasions, she felt confident that it helped to prevent hypoglycaemia:

He’s quite independent and I try to let him do everything and not restrict it because of anything. So just hanging around with his mates, having fun … And on occasions when he’s out and going low, it’s giving him what he needs, it drops small amounts [of insulin] that you can see on the thing [handset], where it’s dropping tiny amounts to keep him level.

#### Negative impacts of using a closed-loop

While all adolescents indicated that the closed-loop had helped them continue to lead mainly normal lives, some also reported feeling self-conscious if the component parts of the system were visible to others (e.g., when they protruded underneath clothing), or when alarms sounded. This included Clare (13 yrs), who described attempting to hide equipment to avoid such situations from happening: “if I’m wearing a dress, I might have to put it [pump] on the back part of my dress so no-one can see it, cause it looks a bit odd”. Others, such as Caitlin (12 yrs), described disliking the attention she received if alarms went off in public settings: “if it’s alarming, when I get it out in class, everyone’s looking at me”. As several young people noted, these concerns could result in them choosing to wear loose-fitting clothing or stopping doing activities such as swimming: “it [CGM sensor] looks a bit like different and weird, especially if you’re like swimming and stuff, so I don’t do that at the moment” (Matthew_15yrs). Parents also described how their child sometimes chose to disconnect from the handset and manually adjust insulin doses when playing sports such as rugby or football, or opted not to take it with them when socialising with friends in order to limit alarms going off in public.

### Few worries about hypoglycaemia

In addition to the daytime benefits highlighted above, most described feeling reassured going to sleep having observed on the handset how the closed-loop kept their glucose levels “flat all night long” (Ben_12yrs). Adolescents also described feeling confident that the closed-loop would suspend insulin delivery and sound an alarm to wake them if they did develop hypoglycaemia when asleep:

it is really good. It’s like if I have a low, it’ll stop the insulin. It’ll just stop giving me it. So I think yeah, it warns me if I’m low as well. … there’s been a few times where I’ve been asleep. And it’s bleeped [alarmed] and I haven’t heard it. And then it would bleep [alarm] louder, and I’d wake up. (Emma_14yrs)

Similarly, parents reported having few worries about hypoglycaemia and rarely experiencing disruptions to sleep because their child’s glucose levels remained stable overnight and they felt confident that the closed-loop would suspend insulin delivery or sound an alarm if necessary. Parents also described not needing to disturb their child in order to perform invasive finger-pricks to check their blood glucose at night because they could do so by consulting the levels displayed on the handset: “I love that graph … I love the fact that I can go in and look at it at night without waking him up, to see what’s going on. That’s really good” (Margaret).

### Adaptation to having diabetes

#### Using closed-loop data to inform self-management decisions

Participants’ accounts also illustrated how using the closed-loop benefitted both individuals who wanted to be in control of managing their diabetes and those who preferred to distance themselves from the condition. Parents of adolescents in the former group reported how, like many in their age group, their child had grown up with, and was adept at using, smartphones and/or other technologies (e.g. fitness trackers, computers, gaming consoles) and, hence, how using such technologies was an integral part of these young people’s self-identities: “It’s bred into them now. It’s common knowledge for them with anything on a phone” (Iris). As parents described, adolescents who had a lifelong familiarity with using these types of technology were also very quick to explore and become comfortable using and navigating their way around the handset and pump screen interfaces.

Similarly, adolescents, such as Annie (16 yrs), described finding the closed-loop technology straightforward to operate and how it enabled her to quickly check her data to determine “where I’m going wrong and what I’m doing right”. As Annie also explained, having access to data on the handset, which included a regularly updated stream of glucose readings and insulin delivered, was both empowering and motivational:

there’s like a green marked area that I should be trying to stay within. It’s like a target zone. So I sometimes scroll through that to see if I’m staying within it. I look at the 24 hour one quite a lot to see if it’s a wonky line or a straight line. I’m quite proud when it’s a straight line.

As well as finding this information motivational, many adolescents described how reviewing their data enabled them to take an active role when managing their diabetes. Most typically, participants described using their data to inform decisions to instruct the pump to administer additional insulin to bring down high glucose faster than the closed-loop was able to do. This included Robert (12yrs), who described choosing to intervene in response to information provided by the CGM because high glucose readings often left him feeling unwell and thirsty:

when me pump says, you know, I’m on a high, over 13, I would have a look on Florence [graph on handset] … as it says the insulin that’s went through then … It helps me to see if I’m still on a high and I need to put in a dose … So like I’ll put in a correction dose just to help it, and push that more through.

#### Creating distance and not being controlled by diabetes

In other cases, participants noted how the components and automated features of the closed-loop system helped to create distance which limited the feeling of being controlled by having diabetes. As Danny pointed out, using the closed-loop had enabled his daughter to “take a back seat”, while Ben (12 yrs) reported finding his diabetes “easier to manage … it’s just you don’t have to do as much”. More specifically, Karen described how using the CGM had helped her son to minimise his involvement in managing diabetes, because “he can just click a button and look at a screen and instantly you know, get a reading, see his levels”. Furthermore, as her son noted, alongside the benefits of not having to do many finger-pricks, using the closed-loop had helped him to “forget” about having diabetes, because he could rely on the system administering and suspending insulin delivery to address glucose digressions:

I just try to like forget about it [diabetes], and that just kinda makes me feel better … and it [closed-loop] makes me feel so that I don’t always- like I don’t have to check [glucose levels] as much, cause … it also gives me insulin when I’m high and it stops giving me it when I’m low … which makes me able to forget about it [diabetes] more. (Matthew_15yrs)

## Discussion

This study has explored adolescents’ and parents’ experiences of using a closed-loop system for 12 months from diagnosis. As our findings have shown, participants did not experience the kinds of family disagreements and tensions typically reported by those using MDI or pump regimens, such as those around adolescents’ dietary choices, or need to perform finger-pricks.^8,9,11–14^ Hence, our findings lend empirical support to studies involving prospective users of closed-loop systems and their family members who expressed hopes that this technology would lessen the burden and stress of managing diabetes, and improve family relationships.^32,33^ The absence/reduction of family disagreements and tensions is a particularly encouraging finding given that, as others have noted, successful diabetes management in youth is often contingent upon parental support, but only if this support can be offered in ways that avoid diabetes-specific family conflict.^34^ One of the reasons for reduced family conflict, as our findings suggest, is the dietary permissiveness facilitated by the closed-loop.^23,35,36^ Indeed, it has been argued that closed-loop technology is especially well-suited to youths because this age group, in particular, is likely to neglect dietary-related self-management tasks, such as counting carbohydrates accurately and administering insulin at mealtimes.^37^

Alongside reduced family conflict, a central benefit of the closed-loop system, highlighted by both parents and adolescents, was its ability to help users lead lives which were not overly disrupted by having diabetes. Unlike adolescents using MDI or pump regimens who often neglect diabetes tasks to fit in with peers,^8,10,12,13,15^ adolescents using the closed-loop reported benefiting from not needing to undertake invasive and inconvenient self-management tasks (e.g. frequent finger-prick tests), and from the system’s ability to automatically adjust insulin delivery in response to rising or falling glucose levels. This meant that adolescents felt confident and able to do ‘normal’ teenage activities, such as socialising with their peers, without worrying about hyperglycaemia and the embarrassment resulting from becoming confused or collapsing in front of others. Similar benefits were noted by parents, who described allowing their child to go out unsupervised because of the glycaemic safety offered by the closed-loop system. Such glycaemic benefits also extended to night-time use, with both youths and parents reporting few worries about hypoglycaemia or disruptions to sleep as a result of the system’s ability to keep glucose levels stable overnight, as others have similarly noted.^22,25^ Indeed, a key finding of this study, is how closed-loop technology has the potential to lessen the biographically disruptive impact of diabetes by virtue of its ability to enable adolescents to eat the same kinds of foods and undertake the same kind of activities as adolescent peers who did not have diabetes. Indeed, there is even potential for use of a closed-loop system to be biographically reinforcing^38^ for those young people who were confident and adept at using technology more generally and who saw technology use as an integral and normal part of being an adolescent.

However, in keeping with findings from studies which explored the views of prospective users of closed-loop systems,^33^ and those involving young people using wearable medical devices,^39–41^ we found that adolescents reported some burdens to using a closed-loop, particularly those who expressed concerns about devices being visible to others. Some adolescents, like adult pump users,^42^ described strategies for ‘passing’^43^ as normal in public and thereby limiting opportunities for enacted stigma;^44^ for example, by wearing loose-fitting clothes or avoiding swimming. Similarly, others attempted to maintain a ‘normal’ identity by disconnecting their devices or not carrying the handset in order to prevent alarms going off in public. While the latter actions risk compromising blood glucose control, they resonate with findings from literature which suggest that people living with diabetes and other chronic conditions often seek a balance between the requirements and demands of self-management tasks with a desire to lead a normal life.^45,46^

Whilst many adolescents, like adult users of closed-loop technology, embraced opportunities to collaborate with the system (e.g. by administering correction doses of insulin) in order to fine-tune and optimise their blood glucose control,^24,27^ others welcomed being able to step back and allow the closed-loop to manage insulin delivery on their behalf. This suggests that closed-loop technology could offer clinical and quality-of-life benefits to individuals who adapt in different ways to having diabetes, including those who prioritise and those who engage less in self-management tasks.^10,13,15^

A key study strength is that it involved a diverse sample of newly-diagnosed adolescents as well as their parents, previously unknown to health professionals; hence, the findings are likely to be more generalisable than those from previous studies where samples have tended to be heavily skewed towards well-educated and highly motivated individuals (e.g.^22,25–27^) However, it is also possible that individuals who opted into the trial were more willing to wear the study devices. Hence, as others have shown,^47^ some young people might find using this technology more burdensome and possibly stigmatising due to the potential visibility of the devices to others. As closed-loop technology is rapidly evolving^3,48^ and our study only focused on users of one particular system, the findings may not be generalisable to other/newer systems.

## Conclusions

Alongside trial data which has demonstrated glycaemic benefits to using a closed-loop system,^49^ our study has highlighted the potential for this kind of technology to reduce or even ameliorate the biographically disruptive impact of diabetes on both youths’ and also parents’ lives. Future developments in closed-loop technology^3,48^ may lessen the biographically disruptive impact of diabetes even further. This includes the integration of the algorithm into an app that can be used on a smartphone or smartwatch, which will overcome the requirement to carry an additional phone handset thus reducing the need for parents to nag their child about keeping devices in close proximity,^3^ together with devices which do not require calibration.^48^

## References

[1] Pettitt DJ Talton J Dabelea D, et al.; for the SEARCH for Diabetes in Youth Study Group. Prevalence of diabetes in US youth in 2009: the SEARCH for diabetes in youth study. Diabetes Care 2014; 37: 402–408. 24041677 10.2337/dc13-1838 PMC3898760

[2] American Diabetes Association. Introduction: standards of medical care in diabetes – 2019. Diabetes Care 2019; 42: S1–S2. 30559224 10.2337/dc19-Sint01

[3] Trevitt S Simpson S Wood A. Artificial pancreas device systems for the closed-loop control of type 1 diabetes: what systems are in development? J Diabetes Sci Technol 2016; 10: 714–723. 26589628 10.1177/1932296815617968 PMC5038530

[4] Bekiari E Kitsios K Thabit H, et al. Artificial pancreas treatment for outpatients with type 1 diabetes: systematic review and meta-analysis. BMJ 2018; 361: k1310. 29669716 10.1136/bmj.k1310 PMC5902803

[5] Laffel LM Connell A Vangsness L, et al. General quality of life in youth with type 1 diabetes: relationship to patient management and diabetes-specific family conflict. Diabetes Care 2003; 26: 3067–3073. 14578241 10.2337/diacare.26.11.3067

[6] Ziegler R Heidtmann B Hilgard D, et al.; for the DPV-Wiss-Initiative. Frequency of SMBG correlates with HbA1c and acute complications in children and adolescents with type 1 diabetes. Pediatr Diabetes 2011; 12: 11–17. 20337978 10.1111/j.1399-5448.2010.00650.x

[7] Hood KK Peterson CM Rohan JM, et al. Association between adherence and glycemic control in pediatric type 1 diabetes: a Meta-analysis. Pediatrics 2009; 124: e1171–e1179. 19884476 10.1542/peds.2009-0207

[8] Spencer JE Cooper HC Milton B. The lived experiences of young people (13–16 years) with type 1 diabetes mellitus and their parents – a qualitative phenomenological study. Diabet Med 2013; 30: e17–e24. 22998426 10.1111/dme.12021

[9] Schilling LS Knafl KA Grey M. Changing patterns of self-management in youth with type I diabetes. J Pediatr Nurs 2006; 21: 412–424. 17101399 10.1016/j.pedn.2006.01.034

[10] Dickinson JK O'Reilly MM. The lived experience of adolescent females with type 1 diabetes. Diabetes Educ 2004; 30: 99–107. 14999898 10.1177/014572170403000117

[11] Davidson M Penney ED Muller B, et al. Stressors and self-care challenges faced by adolescents living with type 1 diabetes. Appl Nurs Res 2004; 17: 72–80. 15154119 10.1016/j.apnr.2004.02.006

[12] Castensøe-Seidenfaden P Teilmann G Kensing F, et al. Isolated thoughts and feelings and unsolved concerns: adolescents’ and parents’ perspectives on living with type 1 diabetes – a qualitative study using visual storytelling. J Clin Nurs 2017; 26: 3018–3030. 27865017 10.1111/jocn.13649

[13] Chilton R Pires-Yfantouda R. Understanding adolescent type 1 diabetes self-management as an adaptive process: a grounded theory approach. Psychol Health 2015; 30: 1486–1504. 26084198 10.1080/08870446.2015.1062482

[14] Ersig AL Tsalikian E Coffey J, et al. Stressors in teens with type 1 diabetes and their parents: immediate and long-term implications for transition to self-management. J Pediatr Nurs 2016; 31: 390–396. 26831378 10.1016/j.pedn.2015.12.012

[15] Commissariat PV Kenowitz JR Trast J, et al. Developing a personal and social identity with type 1 diabetes during adolescence: a hypothesis generative study. Qual Health Res 2016; 26: 672–684. 26893304 10.1177/1049732316628835 PMC9639364

[16] King KM King PJ Nayar R, et al. Perceptions of adolescent patients of the “lived experience” of type 1 diabetes. Diabetes Spectr 2017; 30: 23–35. 28270712 10.2337/ds15-0041 PMC5309904

[17] Rising Holmström M Häggström M Audulv Å, et al. Å. To integrate and manage diabetes in school: youth’s experiences of living with type 1 diabetes in relation to school – a qualitative study. Int Diabetes Nurs 2017; 14: 46–51.

[18] Macaulay GC Boucher SE Yogarajah A. Sleep and night-time caregiving in parents of children and adolescents with type 1 diabetes mellitus – a qualitative study. Behav Sleep Med 2019; 4: 1–5. Aug 10.1080/15402002.2019.1647207 31370700

[19] Bergner EM Williams R Hamburger ER, et al. Sleep in teens with type 1 diabetes: perspectives from adolescents and their caregivers. Diabetes Educ 2018; 44: 541–548. 30193548 10.1177/0145721718799086 PMC6460925

[20] Bury M. Chronic illness as biographical disruption. Sociol Health Illn 1982; 4: 167–182. 10260456 10.1111/1467-9566.ep11339939

[21] Sanders T Elliott J Norman P, et al. Disruptive illness contexts and liminality in the accounts of young people with type 1 diabetes. Sociol Health Illn 2019; 41: 1289–1304. 30968432 10.1111/1467-9566.12906

[22] Barnard KD Wysocki T Allen JM, et al. Closing the loop overnight at home setting: psychosocial impact for adolescents with type 1 diabetes and their parents. BMJ Open Diabetes Res Care 2014; 2: e000025. 10.1136/bmjdrc-2014-000025 PMC4212573 25452866

[23] Iturralde E Tanenbaum ML Hanes SJ, et al. Expectations and attitudes of individuals with type 1 diabetes after using a hybrid closed-loop system. Diabetes Educ 2017; 43: 223–232. 28340542 10.1177/0145721717697244 PMC7162535

[24] Tanenbaum ML Iturralde E Hanes SJ, et al. Trust in hybrid closed-loop among people with diabetes: perspectives of experienced system users. J Health Psychol 2020; 25: 429–438. 28810490 10.1177/1359105317718615 PMC7162558

[25] Barnard KD Wysocki T Ully V, et al. Closing the loop in adults, children and adolescents with suboptimally controlled type 1 diabetes under free living conditions: a psychosocial substudy. J Diabetes Sci Technol 2017; 11: 1080–1088. 28367636 10.1177/1932296817702656 PMC5951034

[26] Lawton J Blackburn M Allen J, et al.; APCam11 Consortium. The impact of using a closed-loop system on food choices and eating practices amongst people with type 1 diabetes: a qualitative study involving adults, teenagers and parents. Diabet Med 2019; 36: 753–760. 30575114 10.1111/dme.13887 PMC6510609

[27] Lawton J Blackburn M Allen J, et al. Participants’ experiences of, and views about, daytime use of a hybrid closed-loop system in real life settings: longitudinal qualitative study. Diabetes Technol Ther 2019; 21: 119–127. 30720338 10.1089/dia.2018.0306 PMC6434584

[28] Boughton C, Allen JM, Tauschmann M, et al. Assessing the effect of closed-loop insulin delivery from onset of type 1 diabetes in youth on residual beta-cell function compared to standard insulin therapy (CLOuD study): a randomised parallel study protocol. *BMJ Open* 2020;10: e033500. 10.1136/bmjopen-2019-033500 PMC7069267 32169925

[29] Pope C Mays N. Qualitative research: reaching the parts other methods cannot reach: an introduction to qualitative methods in health and health services research. BMJ 1995; 311: 42–45. 7613329 10.1136/bmj.311.6996.42 PMC2550091

[30] Strauss A Corbin JM. Basics of qualitative research: grounded theory procedures and techniques. Thousand Oaks, CA: Sage Publications, Inc, 1990.

[31] May C Finch T. Implementing, embedding, and integrating practices: an outline of normalization process theory. Sociology 2009; 43: 535–554.

[32] Garza KP Jedraszko A Weil LE, et al. Automated insulin delivery systems: hopes and expectations of family members. Diabetes Technol Ther 2018; 20: 222–228. 29565721 10.1089/dia.2017.0301 PMC6422006

[33] Naranjo D Suttiratana SC Iturralde E, et al. What end users and stakeholders want from automated insulin delivery systems. Diabetes Care 2017; 40: 1453–1461. 28842523 10.2337/dc17-0400 PMC5864142

[34] Feldman MA Anderson LM Shapiro JB, et al. Family-based interventions targeting improvements in health and family outcomes of children and adolescents with type 1 diabetes: a systematic review. Curr Diab Rep 2018; 18: 15. 29457190 10.1007/s11892-018-0981-9

[35] Elleri D Maltoni G Allen JM, et al. Safety of closed-loop therapy during reduction or omission of meal boluses in adolescents with type 1 diabetes: a randomized clinical trial. Diabetes Obes Metab 2014; 16: 1174–1178. 24909206 10.1111/dom.12324 PMC4192111

[36] Cherñavvsky DR Deboer MD Keith-Hynes P, et al. Use of an artificial pancreas among adolescents for a missed snack bolus and an underestimated meal bolus. Pediatr Diabetes 2016; 17: 28–35. 25348683 10.1111/pedi.12230

[37] Sherr JL. Closing the loop on managing youth with type 1 diabetes: children are not just small adults. Diabetes Care 2018; 41: 1572–1578. 29936422 10.2337/dci18-0003 PMC6054496

[38] Carricaburu D Pierret J. From biographical disruption to biographical reinforcement: the case of HIV‐positive men. Sociol Health Illness 1995; 17: 65–88.

[39] Messer LH Johnson R Driscoll KA, et al. Best friend or spy: a qualitative meta‐synthesis on the impact of continuous glucose monitoring on life with type 1 diabetes. Diabet Med 2018; 35: 409–418. 29247556 10.1111/dme.13568

[40] Alsaleh FM Smith FJ Taylor KM. Experiences of children/young people and their parents, using insulin pump therapy for the management of type 1 diabetes: qualitative review. J Clin Pharm Ther 2012; 37: 140–147. 21729118 10.1111/j.1365-2710.2011.01283.x

[41] Lawton J Blackburn M Allen J, et al. Patients’ and caregivers’ experiences of using continuous glucose monitoring to support diabetes self-management: qualitative study. BMC Endocr Disord 2018; 18: 12. 29458348 10.1186/s12902-018-0239-1 PMC5819241

[42] Hess A. In/visible personal medical devices: the insulin pump as a visual and material mediator between selves and others. In: Lynch R Farrington C (eds) Quantified lives and vital data: exploring health and technology through personal medical devices. London: Palgrave Macmillan, 2018, pp.71–96.

[43] Goffman E. The presentation of self in everyday life. London: Harmondsworth, 1978.

[44] Scambler G Hopkins A. Being epileptic: coming to terms with stigma. Sociol Health Illness 1986; 8: 26–43.

[45] Paterson BL Thorne S Dewis M. Adapting to and managing diabetes. Image J Nurs Sch 1998; 30: 57–62. 9549943 10.1111/j.1547-5069.1998.tb01237.x

[46] Campbell R Pound P Pope C, et al. Evaluating meta-ethnography: a synthesis of qualitative research on lay experiences of diabetes and diabetes care. Soc Sci Med 2003; 56: 671–684. 12560003 10.1016/s0277-9536(02)00064-3

[47] Farrington C Allen J Tauschmann M, et al. Factors affecting recruitment of participants for studies of diabetes technology in newly diagnosed youth with type 1 diabetes: a qualitative focus group study with parents and children. Diabetes Technol Ther 2016; 18: 568–573. 27355100 10.1089/dia.2016.0157 PMC5035376

[48] Allen N Gupta A. Current diabetes technology: striving for the artificial pancreas. Diagnostics 2019; 9: 31. 10.3390/diagnostics9010031 PMC6468523 30875898

[49] Karageorgiou V Papaioannou TG Bellos I, et al. Effectiveness of artificial pancreas in the non-adult population: a systematic review and network meta-analysis. Metabolism 2019; 90: 20–30. 30321535 10.1016/j.metabol.2018.10.002

